# Importance of continuous treatment with intravitreal aflibercept injections in patients with neovascular age-related macular degeneration—12-month post hoc analysis of the PERSEUS real-world evidence study

**DOI:** 10.1007/s00417-020-04803-8

**Published:** 2020-08-13

**Authors:** Joachim Wachtlin, Nicole Eter, Zoran Hasanbasic, Georgios Keramas, Christine Rech, Helmut Sachs, Harald Schilling, Peter Wiedemann, Carsten Framme

**Affiliations:** 1 Abteilung für Augenheilkunde, Sankt Gertrauden-Krankenhaus, Paretzer Straße 12, 10713 Berlin, Germany; 2 MHB, Medizinische Hochschule Brandenburg, Neuruppin, Germany; 3 Klinik für Augenheilkunde, Universitätsklinikum Münster, Münster, Germany; 4 Medizinische Fachabteilung, Bayer Vital GmbH, Leverkusen, Germany; 5 Data Generation, Bayer Vital GmbH, Leverkusen, Germany; 6 Augenklinik, Städtisches Klinikum Dresden-Friedrichstadt, Dresden, Germany; 7 Klinik für Augenheilkunde, St.-Johannes-Hospital, Dortmund, Germany; 8 Klinik und Poliklinik für Augenheilkunde, Universitätsklinikum Leipzig, Leipzig, Germany; 9 Klinik für Augenheilkunde, Medizinische Hochschule Hannover, Hannover, Germany

**Keywords:** Neovascular age-related macular degeneration, Intravitreal aflibercept, Intravitreal injection, Visual acuity

## Abstract

**Purpose:**

To investigate the influence of treatment regularity with intravitreal aflibercept injections (IVT-AFL injections) on visual acuity (VA) outcomes in patients with neovascular age-related macular degeneration (nAMD) enrolled in the PERSEUS trial who received at least 7 IVT-AFL injections during the first year.

**Methods:**

This was a post hoc analysis of the PERSEUS trial, a prospective, non-interventional, multicenter cohort study, and included 370 patients with nAMD who had received ≥ 7 IVT-AFL injections during year 1. In addition to the prespecified subgroups of treatment-naïve and previously treated patients, results were compared between patients with regular (*n* = 209) and irregular (*n* = 161) treatment. Regular treatment was defined as initial dosing with monthly IVT-AFL injections for 3 months, then bimonthly IVT-AFL injections until month 12. Irregular treatment was defined as any deviation from regular treatment (provided ≥ 7 injections were received). The outcome of primary interest was the mean change in VA from baseline after 12 months. Further outcomes of interest included VA gain or loss, proportion of patients achieving reading vision, and percentage of patients with fluid.

**Results:**

At month 12, the mean (± standard deviation, SD) VA improvement from baseline was 6.1 ± 15.6 Early Treatment Diabetic Retinopathy Study letters in the regular cohort and 2.5 ± 16.7 letters in the irregular cohort with ≥ 7 IVT-AFL injections (*P* = 0.0514). Best results were obtained in the treatment-naïve regular sub-cohort with a mean ± SD VA improvement of 8.0 ± 17.7 letters, whereas treatment-naïve patients with irregular treatment experienced a considerably lower VA gain (2.8 ± 20.0 letters). Irregular treatment consistently correlated with inferior results in treatment-naïve patients. At month 12, the proportion of treatment-naïve patients who had experienced a worsening of ≥ 5 letters was 29.6% in the irregular sub-cohort versus 13.6% in the regular sub-cohort (*P* = 0.0049). However, among the treatment-naïve patients, the mean number of injections was significantly higher in the irregular than in the regular sub-cohort (8.0 ± 1.2 vs. 7.4 ± 0.6; *P* = 0.0001). Furthermore, compared with the treatment-naïve, regular sub-cohort, patients in the irregular sub-cohort had more visits (19.1 ± 8.6 vs. 16.1 ± 5.7), VA tests (14.2 ± 6.9 vs. 12.0 ± 4.6), and optical coherence tomography examinations (5.1 ± 3.7 vs. 3.4.0 ± 3.0).

**Conclusions:**

Although irregularly treated patients received more injections and more monitoring visits during the first year of IVT-AFL treatment, they experienced worse VA outcomes than regularly treated patients.

**Electronic supplementary material:**

The online version of this article (10.1007/s00417-020-04803-8) contains supplementary material, which is available to authorized users.

## Introduction

Neovascular age-related macular degeneration (nAMD), a chronic progressive disease mainly affecting the elderly, is one of the most common causes of visual impairment and blindness in developed countries [1]. Choroidal neovascularization is driven by overexpression of vascular endothelial growth factor (VEGF) and is associated with rapid loss of visual acuity (VA). Therefore, early visual stabilization appears crucial in order to preserve as much VA as possible over the course of time [2].

Anti-VEGF agents for intravitreal injection represented the first therapeutic option to improve VA instead of merely slowing down the rate of vision loss. Their development is thus considered to be a milestone in the treatment of nAMD [3].

One of the first anti-VEGF agents to be approved for nAMD was ranibizumab. In the phase 3 MARINA study, treatment with monthly intravitreal ranibizumab injections over 24 months improved visual and anatomic outcomes [4]. However, subsequent observational studies with ranibizumab have consistently shown that vision outcomes obtained in real-world conditions did not match the results from controlled clinical trials. This coincided with a lower number of injections than expected as compared with a comparable time period under controlled trial conditions [5–8].

Intravitreal aflibercept (IVT-AFL, Regeneron, Tarrytown, NY, and Bayer HealthCare, Berlin, Germany) has a substantially greater binding affinity to VEGF than ranibizumab [9]. In the VIEW 1 and VIEW 2 studies, bimonthly IVT-AFL injections following 3 initial monthly doses significantly improved VA and visual and anatomic outcomes were similar to monthly ranibizumab in terms of visual and anatomic outcomes [10].

In order to examine the efficacy of IVT-AFL injections in patients with nAMD under real-life conditions in Germany, the PERSEUS study (PERSEUS = Prospective Non-interventional Study to Assess the Effectiveness of Intravitreal Aflibercept in Routine Clinical Practice in Patients with Neovascular Age-Related Macular Degeneration) was carried out between 2013 and 2017. In a population of 848 patients, VA changes and treatment patterns were observed over 24 months [11]. At the time of enrolment, the European Summary of Product Characteristics (SPC) [12] specified treatment with IVT-AFL injections as follows: initial dosing with monthly injections of 2 mg IVT-AFL for three consecutive doses followed by injections of 2 mg IVT-AFL every 2 months throughout the first year. Thus, patients should receive a minimum of 7 IVT-AFL injections within the first 12 months.

In the 12-month analysis of the PERSEUS study, treatment-naïve patients achieved a VA gain of 5.3 letters, whereas patients who had received previous nAMD treatment with other medications (previously treated patients) tended to retain their vision (− 0.1 letters, *P* < 0.0001) [11].

Analysis of treatment patterns revealed that nearly three-quarters (73.9%) of the total study population (treatment-naïve and previously treated patients) deviated from the recommended treatment scheme. Those were defined as irregularly treated patients. Furthermore, stratified analysis demonstrated a major impact of treatment patterns on VA outcome. For instance, treatment-naïve patients who received IVT-AFL injections at regular intervals achieved a VA gain of 8.0 letters, which comes close to the VA gains reported in RCTs [10, 11]. Deviations from the treatment scheme in treatment-naïve patients were associated with a lower VA gain of 4.0 letters at month 12 [11].

In the total PERSEUS study population, patients in the irregular cohort received fewer injections than patients in the regular cohort (5.2 vs. 7.5 IVT-AFL injections). However, irregular treatment could not solely be attributed to an insufficient number of IVT-AFL injections since almost one-third of the irregular cohort had still received ≥ 7 IVT-AFL injections during the first year. This population is of particular interest because it provides the opportunity to evaluate the influence of treatment regularity on the success of IVT-AFL therapy. Thus, we conducted a post hoc analysis to compare the 12-month outcomes of irregularly treated patients who received at least 7 IVT-AFL injections in the first year with those of regularly treated patients.

## Methods

### Study design

This is a post hoc analysis of the PERSEUS study, the methods for which have been published previously [11]. Briefly, PERSEUS was a prospective, non-controlled, non-interventional, multicenter cohort study conducted in 66 study sites in Germany. Patients were enrolled consecutively from July 2013 to March 2015 and followed for up to 24 months. All treatment decisions, including the decision to treat with IVT-AFL, were made by the treating physician, independently of study participation. Ethics approval was obtained from the respective independent ethics committees or institutional review boards and all participants provided written informed consent.

### Eligibility

In this post hoc analysis, only patients who received at least 7 IVT-AFL injections during the first 12 months of treatment were included. Data were stratified for treatment-naïve and previously treated patients and regular and irregular treatment. Regular treatment was defined as initial dosing with monthly 2 mg IVT-AFL for 3 months (− 1/+ 2 weeks), followed by bimonthly 2 mg IVT-AFL (− 2/+4 weeks). This treatment regimen is in accordance with the European SPC but allows for real-life clinical practice flexibility. Regular treatment results in at least 7 (up to 9, due to the allowed time windows) IVT-AFL injections during the first year. Any treatment differing from the licensed posology for IVT-AFL with its given injection intervals (including the allowed time windows) was considered to be irregular, including both less-frequent and more-frequent dosing. In accordance with the German SPC at the time of the study, monitoring between injections was not obligatory but was subject to the judgement of the attending physician.

Inclusion and exclusion criteria of the PERSEUS study are described in the publication of the 12-month interim analysis [11]. Briefly, patients with nAMD treated with IVT-AFL in accordance with the national SPC were eligible for the PERSEUS study. Exclusion criteria were as listed in the national SPC. Additionally, patients with scarring, fibrosis, or atrophy comprising the foveal center, or who were treated for nAMD with any other agent in the study eye, were excluded. Eyes with retinal pigment epithelium tears, detachment, or lesion of the retinal pigment epithelium were eligible. Previous nAMD treatment, including anti-VEGF agents (ranibizumab, bevacizumab, pegaptanib), was permitted.

### Objectives

The outcome of primary interest of this post hoc analysis was the mean change in VA from baseline. VA assessment and conversion to Early Treatment Diabetic Retinopathy Study (ETDRS) letter score have been described previously [11]. Key secondary outcomes of interest included monitoring of treatment patterns. For this purpose, data on the number of injections, visits, optical coherence tomography (OCT) measurements (e.g., proportion of patients with no fluid after 4 and 12 months), and VA measurements were analyzed. In addition, the mean time between injections was calculated.

### Statistical analyses

Due to the observational nature of this study, there was no predefined visit schedule. Changes from baseline in VA were analyzed for time points equivalent to months 1 and 2, as well as months 4, 6, 8, 10, and 12. Until month 2 (treatment initiation), a window of ± 15 days was allowed for all time points. For the subsequent maintenance phase, the window was expanded to ± 30 days. Therefore, the number of patients varied for different end points. The last observation carried forward (LOCF) approach (in this case, the last observation refers to the last VA measurement) was applied to impute missing values after the first 120 days after the first IVT-AFL injection. The Wilcoxon rank-sum test was used to compare VA changes between patient cohorts. Linear regression was performed in order to examine the association between selected covariates and change in VA letter score between baseline and follow-up after 12 months. In addition, all independent covariates (baseline VA letter score, age at indication, gender, pre-treatment, and regular and irregular treatment defined by deviations from the treatment scheme as specified in the national SPC) were entered into a stepwise multivariate linear regression. The entry level was *P* = 0.5 and the stay level was *P* = 0.05. All remaining significant covariates were considered to be associated with the change in VA letter score after 12 months. In order to avoid varying sample sizes, missing observations were dropped for univariate and multivariate regression. In further analyses, logistic regression was applied to examine the association between baseline covariates and regular or irregular treatment. In order to determine the association, univariate logistic regression was performed for the dependent variable regular or irregular treatment with the outcomes of regular or irregular injections. Afterwards, all independent covariates (baseline VA letter score, age at indication, gender, and previous treatment) were entered into a stepwise multivariate logistic regression for the above-mentioned dependent variable. The entry level was *P* = 0.5 and the stay level was *P* = 0.05. All *P* values given in this post hoc analysis are purely descriptive in nature; no formal predefined hypothesis was confirmed or rejected.

## Results

### Patient characteristics

The PERSEUS study enrolled 848 patients. In this 12-month post hoc analysis, 370 patients who had received ≥ 7 IVT-AFL injections during the first year of treatment were included. Among those, 209 (56.5%) were regularly treated (regular cohort), while 161 (43.5%) deviated from the treatment scheme that was specified by the European SPC (irregular cohort). In the post hoc analysis, 211 patients were treatment-naïve (regular sub-cohort: *n* = 130; irregular sub-cohort: *n* = 81), whereas 159 had received previous nAMD treatment (regular sub-cohort: *n* = 79; irregular sub-cohort: *n* = 80) (Table 1).

**Table 1:** Baseline characteristics of patients with ≥ 7 IVT-AFL injections during the first 12 months of IVT-AFL treatment

	Regular cohort	Irregular cohort (≥ 7 IVT-AFL injections)
Total population (*n* = 370), *n* (%)	209 (56.6)	161 (43.5)
Mean age (SD), years	77.9 (7.5)	76.4 (8.0)
Female, *n* (%)	131 (62.7)	88 (54.7)
Mean retinal thickness, μm	351.4	348.7
Baseline mean VA letter score (SD)*	53.6 (17.4)	54.0 (18.6)
Treatment-naϊve patients (*n* = 211), *n* (%)	130 (61.1)	81 (38.4)
Mean age (SD), years	77.2 (7.3)	75.4 (7.8)
Female, *n* (%)	79 (60.8)	47 (58.0)
Mean retinal thickness, μm	360.1	372.3
Baseline mean VA letter score (SD)*	52.8 (17.5)	55.2 (17.8)
Previously treated patients (*n* = 159), *n* (%)	79 (49.7)	80 (50.3)
Mean age (SD), years	79.0 (7.7)	77.4 (8.1)
Female, *n* (%)	52 (65.8)	41 (51.3)
Mean retinal thickness, μm	339.2	324.3
Baseline mean VA letter score (SD)*	54.9 (17.2)	52.8 (19.5)

*Patients with available data on change at 12 months. *SD* standard deviation, *VA* visual acuity

The mean age of the total post hoc population was 77.2 ± 7.2 years, and 219 patients (59.2%) were female. Table 1 summarizes the baseline characteristics of regularly and irregularly treated patients who had received ≥ 7 IVT-AFL injections. The mean baseline VA was similar between the regular and irregular cohorts (53.6 ± 17.4 letters vs. 54.0 ± 18.6 letters; *P* = 0.6381). Overall, the baseline characteristics appeared to be well balanced in the two groups and were in line with the main study population [11].

Fifty patients discontinued treatment during the first 12 months of therapy: 18 patients (13.8%) in the treatment-naïve regular sub-cohort, 13 patients (16.0%) in the treatment-naïve irregular sub-cohort, 15 patients (19.0%) in the previously treated regular sub-cohort, and four patients (5.0%) in the previously treated irregular sub-cohort.

Previously treated patients received ranibizumab only (66.7%), off-label bevacizumab only (22.6%), ranibizumab and bevacizumab (8.2%), or other previous treatment (2.5%). The average duration of previous treatment was 17.3 ± 19.8 months.

### Number of injections

During the first 12 months of IVT-AFL treatment, patients in the irregular cohort received a statistically significant higher mean number of injections than patients in the regular cohort (irregular 8.1 ± 1.2 vs. regular 7.5 ± 0.6; *P* < 0.0001). This result was consistent in both treatment-naïve and previously treated patients (Table 2). Table 2 provides a detailed brea7kdown of injection numbers in the regular and the irregular cohorts.

**Table 2:** Mean number of injections and breakdown of injection numbers

	**Mean number of injections (SD)**		
	**Regular cohort**	**Irregular cohort**	***P*** **value**
Total post hoc population) (*N* = 370)	7.5 (0.6) (*n* = 209)	8.1 (1.2) (*n* = 161)	*P* < 0.0001
Treatment-naïve (*N* = 211)	7.4 (0.6) (*n* = 130)	8.0 (1.2) (*n* = 81)	*P* = 0.0001
Pretreated (*N* = 159)	7.5 (0.6) (*n* = 79)	8.2 (1.2) (*n* = 80)	*P* = 0.0001
	**Breakdown of injection numbers**		
**Number of IVT-AFL injections**	**Regular cohort (*n*)** ***N*** **= 209**	**Irregular cohort (*n*)** ***N*** **= 161**	
7	123	58	
8	77	56	
9	9	29	
10	-	12	
11	-	3	
12	-	1	
13	-	2	

*IVT-AFL* intravitreal aflibercept, *SD* standard deviation

### Mean change in visual acuity

Treatment patterns were associated with treatment outcome: Despite a lower mean number of injections, patients receiving regular treatment achieved a higher mean VA improvement than irregularly treated patients (regular cohort 6.1 ± 15.6 letters vs. irregular 2.5 ± 16.7 letters; *P* = 0.0514).

**Fig. 1:**
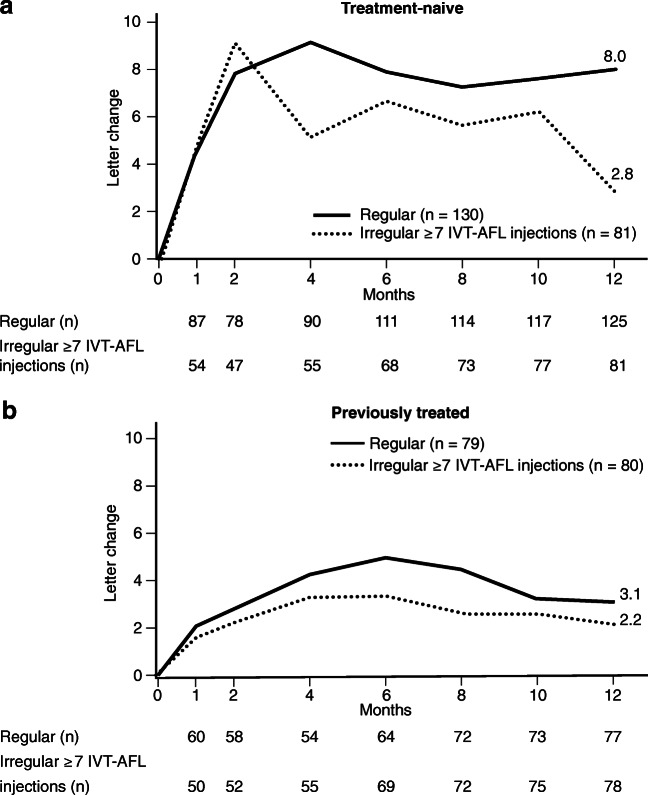
Mean change in VA within the first year in sub-cohorts of treatment-naïve (**a**) and previously treated (**b**) regular and irregular (≥ 7 IVT-AFL injections) patients (LOCF). *IVT-AFL injections*, intravitreal aflibercept injections; *LOCF*, last observation carried forward; *VA*, visual acuity. Adobe Illustrator CC 2020 was used to create the artwork.

This finding was especially pronounced in treatment-naïve patients. While treatment-naïve patients with regular treatment experienced the highest VA gain with 8.0 ± 17.7 letters, those with irregular treatment improved merely by 2.8 ± 20.0 letters (Fig. 1a).

The influence of regular treatment was less distinct in previously treated patients, where mean VA improvement was generally lower than that in treatment-naïve patients (previously treated, regular sub-cohort 3.1 ± 10.7 letters vs. previously treated, irregular sub-cohort 2.2 ± 16.7 letters) (Fig. 1b).

### Mean time between injections

Generally, the mean times between injections were similar for regularly and irregularly treated patients throughout all treatment phases (Table 3). However, the standard deviations were clearly larger in the irregularly treated sub-cohorts, indicating a higher variability in the injection intervals. This was especially striking for the mean time between the 3rd and 4th IVT-AFL, which (according to the European SPC) marks the transition from the initial treatment phase with monthly injections to the maintenance phase with bimonthly injections. In the treatment-naïve, regular sub-cohort, the mean time between the 3rd and the 4th injection was 59.5 ± 7.1 days. In the irregular sub-cohort, the mean time was similar (58.9 days), but with ± 24.5 the SD was triple compared with the regular sub-cohort, indicating that the distribution of the time between injections for irregularly treated patients was spread over a larger range.

**Table 3:** Mean time between injections

		Mean time (d) between first 3 IVT-AFL injections		Mean time (d) between 3rd and 4th IVT-AFL		Mean time (d) between IVT-AFL injections in year 1 starting at 3rd IVT-AFL	
		Mean (d)	SD	Mean (d)	SD	Mean (d)	SD
Treatment-naϊve	Regular	30.5	3.2	59.5	7.1	58.9	3.9
	irregular (≥ 7 IVT-AFL injections)	31.6	8.9	58.9	24.5	55.0	8.7
Previously treated	Regular	30.7	3.0	59.7	8.5	59.5	4.1
	irregular (≥ 7 IVT-AFL injections)	34.1	11.8	61.6	32.6	52.9	9.8

*IVT-AFL* intravitreal aflibercept injection, *d* days, *SD* standard deviation

### Stratification for regular and irregular initial dosing

In the irregularly treated cohort, 64.6% of the patients received initial dosing with 3 monthly injections according to the SPC within the given tolerance (regular initial dosing), whereas 35.7% deviated from this treatment scheme (irregular initial dosing) (data not shown). When analyzed based on regular and irregular initial dosing, VA outcomes at 12 months in the treatment-naïve irregular sub-cohort were 2.3 ± 19.6 letters with regular initial dosing and 3.7 ± 21.0 letters with irregular initial dosing. In the previously treated irregular sub-cohort, VA outcomes were 2.5 ± 13.4 letters with regular initial dosing and 1.6 ± 10.7 letters with irregular initial dosing.

### Other visual acuity end points

**Fig. 2:**
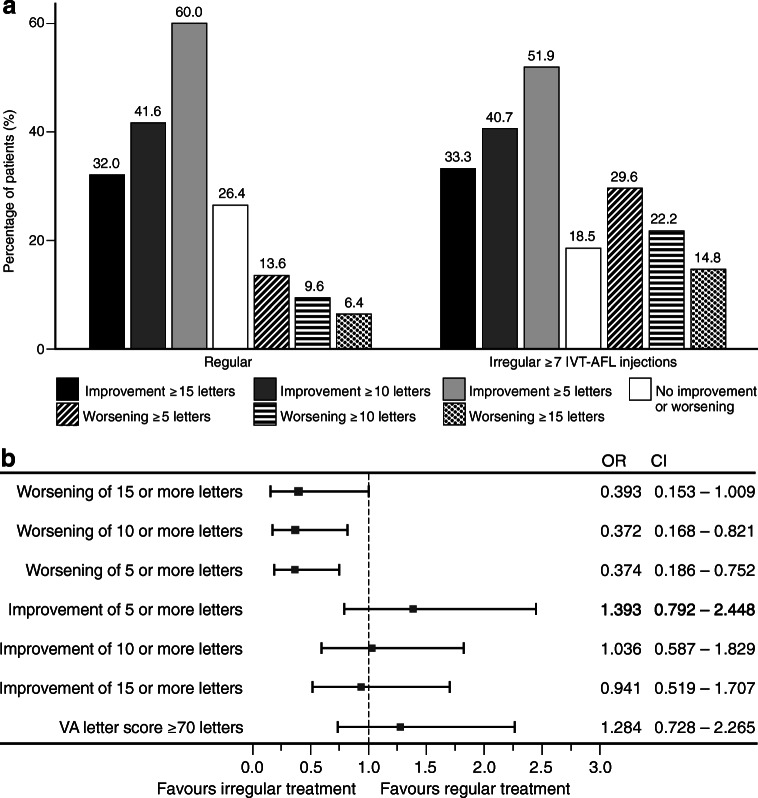
Percentage of treatment-naïve patients with VA gain or loss with regular or irregular (≥ 7 IVT-AFL injections) treatment (LOCF) (**a**). Forest plots illustrating the effect of regular and irregular treatment on improvement or worsening of VA in treatment-naïve patients (LOCF) (**b**). *IVT-AFL*, intravitreal aflibercept; *LOCF*, last observation carried forward; *CI*, confidence interval; *OR*, odds ratio. Adobe Illustrator CC 2020 was used to create the artwork.

Among the treatment-naïve cohort, the proportion of patients that experienced a VA loss was significantly larger with irregular treatment compared with regular treatment (worsening of ≥ 5 letters 29.6% vs. 13.6%, *P* = 0.0049; worsening of ≥ 10 letters 22.2% vs. 9.6%, *P* = 0.0121; worsening of ≥ 15 letters 14.8% vs. 6.4%, *P* = 0.0463 for irregular vs. regular treatment, respectively; Fig. 2a). On the other hand, more patients tended to achieve a VA improvement ≥ 5 letters when treated regularly (Fig. 2a). Similar trends were observed among previously treated patients (see Supplementary Figure 1).

Furthermore, logistic regression analysis revealed that irregular treatment was significantly associated with higher risk of experiencing a VA loss of ≥ 5 and ≥ 10 letters for treatment-naïve patients (Fig. 2b).

**Fig. 3:**
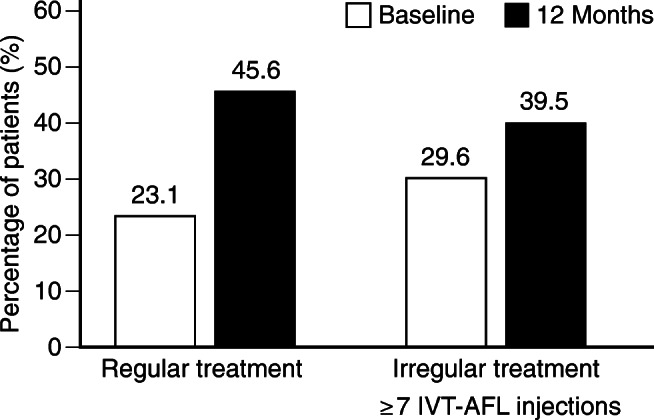
Proportion of treatment-naïve patients with reading vision (VA ≥ 70 letters) at baseline and at 12 months for regular and irregular (≥ 7 IVT-AFL injections) treatment (LOCF). *IVT-AFL*, intravitreal aflibercept; *LOCF*, last observation carried forward; *VA*, visual acuity. Adobe Illustrator CC 2020 was used to create the artwork.

Another crucial patient-relevant outcome is the reading vision (defined as VA ≥ 70 letters, decimal ≈ 0.5 (20/40)). In many countries, it is also the minimal requirement to be legally allowed to drive and thus is referred to as “driving vision.” Among treatment-naïve patients who received regular IVT-AFL treatment, the proportion of patients with a VA ≥ 70 letters increased from 23.1% at baseline to 45.6% at 12 months. In contrast, this proportion only increased from 29.6% at baseline to 39.5% at 12 months in treatment-naïve patients with irregular treatment (Fig. 3). In logistic regression analysis, regular treatment was non-significantly associated with a higher likelihood of achieving reading vision after 12 months (OR 1.284, 95% confidence interval = 0.728–2.265; Fig. 2b).

Among previously treated patients, the influence of irregular treatment on the proportion of patients with a VA ≥ 70 letters was negligible (see Supplementary Figure 2).

### Patients without fluid

**Fig. 4:**
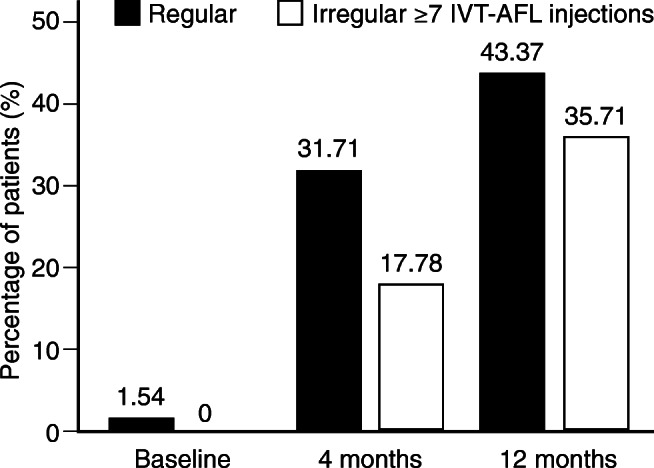
Percentage of treatment-naïve patients without fluid at baseline, 4 months, and 12 months for regular and irregular (≥ 7 IVT-AFL injections) treatment (LOCF). *IVT-AFL*, intravitreal aflibercept injections; *LOCF*, last observation carried forward. Adobe Illustrator CC 2020 was used to create the artwork.

Regular and irregular treatment patterns also affected anatomic outcome measures. The proportion of treatment-naïve patients without fluid (subretinal, intraretinal, or subretinal pigment epithelial) on OCT was larger with regular treatment than with irregular treatment at month 4 (31.71% vs. 17.78%) and at month 12 (43.37% vs. 34.71%) (Fig. 4). Similarly, among previously treated patients, a higher percentage in the regular sub-cohort did not have detectable fluid on OCT (see Supplementary Figure 3).

### Other secondary outcomes

During the first year of treatment, significantly more OCT examinations were performed in the irregular cohort than in the regular cohort (mean number of OCT examinations; 3.9 ± 3.2 vs. 5.2 ± 3.5). Furthermore, numerically more visits, post-injection safety visits, and VA tests were observed in the irregular cohort (Table 4).

**Table 4:** Number of visits and ophthalmological assessments per patient after 12 months

	Treatment-naϊve—regular (*n* = 130)	Treatment-naϊve—irregular (*n* = 81)	Previously treated—regular (*n* = 79)	Previously treated—irregular (*n* = 80)	Regular total (*n* = 209)	Irregular (≥ 7 IVT-AFL injections) total (*n* = 161)
Number of visits*, mean (SD)	16.1 (5.7)	19.1 (8.6)	17.1 (7.7)	18.3 (7.7)	16.5 (6.5)	18.7 (8.1)
Number of post-injection safety visits, mean (SD)	6.0 (4.9)	7.5 (7.8)	6.7 (6.7)	6.6 (6.6)	6.3 (5.7)	7.1 (7.2)
Number of VA tests, mean (SD)	12.0 (4.6)	14.2 (6.9)	13.1 (4.7)	14.1 (6.4)	12.4 (4.7)	14.2 (6.6)
Number of OCTs, mean (SD)	3.4 (3.0)	5.1 (3.7)	4.6 (3.4)	5.3 (3.2)	3.9 (3.2)	5.2 (3.4)

*Visits include injection visits, control visits, combined visits. *IVT-AFL* intravitreal aflibercept, *OCT* optical coherence tomography, *SD* standard deviation, *VA* visual acuity

## Discussion

The PERSEUS study has previously shown a clear association between consistent, regular treatment and beneficial VA outcome [11]. This post hoc analysis provides further evidence that shows that these positive outcomes were likely not driven solely by injection numbers, but rather that consistency and regularity of treatment are also important to achieve optimal VA outcomes in patients with nAMD.

The PERSEUS study was the first major prospective study evaluating IVT-AFL treatment under real-life conditions in German clinical practices and hospitals [11]. In the 12-month analysis of the total study population, irregular treatment was associated with a significantly lower VA gain. While treatment-naïve, regularly treated patients achieved a VA gain of 8.0 letters, irregularly treated patients improved only by 4.0 letters. This was associated with a significantly lower mean number of injections in the irregularly treated cohort (7.4 vs. 5.1 IVT-AFL injections).

Similarly, various observational trials with ranibizumab (AURA [13], COMPASS [7], and OCEAN [14]) have shown that long-term VA outcomes obtained in randomized clinical trials could not be achieved under real-world conditions. The observational trials consistently showed there were fewer injections under real-world conditions than would be expected based on the phase 3 study results [4].

All patients in this PERSEUS post hoc population, however, received ≥ 7 IVT-AFL injections during the first treatment year. Nevertheless, deviations from the recommended treatment scheme were associated with poorer outcomes. Treatment-naïve patients with ≥ 7 IVT-AFL injections but irregular treatment improved only by 2.8 letters at 12 months compared with 8.0 letters in the regularly treated group. Furthermore, irregular treatment influenced other visual and anatomic outcomes, particularly in treatment-naïve patients. Fewer patients in the irregular cohort achieved reading vision (≥ 70 letters) compared with the regular cohort. The risk of vision loss was significantly increased and a worsening of ≥ 5 letters, ≥ 10 letters and ≥ 15 letters was twice as common in the irregular cohort compared with the regular cohort. Fewer patients were diagnosed without fluid on OCT at 4 and 12 months of treatment for the irregular cohort compared with the regular cohort.

Although similar trends were observed in previously treated patients, the difference between regular and irregular treatment was less pronounced. This may be due to the fact that previously treated patients generally experience less functional improvement because structural damage may have progressed more than in treatment-naïve patients. Thus, the limited ability of previously treated patients to improve may “mask” the influence of irregular treatment.

These data suggest that regular treatment can lead to better visual and anatomic outcomes, whereas irregular treatment, even with a similar number of injections, may result in less robust improvement. Interestingly, irregularly treated patients received a modest but statistically significantly higher number of injections than regularly treated patients. For the individual patient, 0.5 injections more per year will most likely not be clinically relevant in terms of visual outcome. The higher injection number in the irregular cohort mainly arises from a higher proportion of patients that received ≥ 9 injections during the first year compared with the regular cohort. Due to the overall low number of patients in this population (*n* = 47), we could not conduct a statistically meaningful analysis on their VA outcome. We may however speculate about the possible reasons for overtreatment.

First, this group may include patients that did not respond well to the IVT-AFL injections and due to persistent disease activity received more-frequent treatment than recommended. Patients that are difficult to treat may tend to do worse and likely contribute to the lower VA gain we observed in the irregularly treated cohort. However, since the change in VA improves similarly during the upload phase in regular and irregularly treated treatment-naïve patients, we do not assume that a high percentage of bad responders is the main reason for the lower VA gain at month 12.

Second, it may include patients whose disease activity increased due to a treatment delay and thus received the following injections in shorter intervals in an attempt to compensate for worsening of the disease. This situation may arise if an upcoming injection was delayed either because no disease activity was detected in OCT according to a pro-re-nata (PRN) scheme or due to non-adherence. We can speculate that patients with delayed treatment are at an increased risk of irreversible photoreceptor damage. This in turn cannot be compensated for by subsequent IVT-AFL injections with higher frequency.

More than 40% of the patients receiving at least 7 injections during the first treatment year in the PERSEUS study were irregularly treated. One factor possibly contributing to this observation may be, that at the time the study was active (2013–2017), German professional associations recommended solely as-needed treatment schemes with anti-VEGF agents. It is thus likely that patients in the irregularly treated cohort were treated according to schemes endorsed by the German ophthalmological societies, including off-label IVT-AFL in a PRN or a treat-and-extend (TAE) regimen [15]. Since monthly OCT controls are recommended within a PRN scheme, this might explain the higher number of visits and examinations in the irregular cohort with ≥ 7 injections during the 12 months of treatment.

On the first glance, the mean time interval between injections appeared numerically similar in the regular and irregular cohorts. However, standard deviations in the irregular cohorts were clearly larger, indicating a higher variance in treatment intervals. This was especially pronounced between the 3rd and 4th injections, when the upload phase with three monthly injections is completed and treatment proceeds to the maintenance phase. This finding might also be associated with treatment according to PRN (or TAE) schemes since the switch to those regimens is usually performed following the initial three injections. Especially with PRN schemes, a rather long phase without an injection following the upload phase is common. At this stage of treatment, it is important that patients and physicians are aware of the chronicity of the disease and that a VA improvement at the end of the upload phase may be lost with insufficient retreatment.

Another possible reason for irregular treatment is insufficient adherence. The recently published PONS study [16] showed that non-persistence (no contact with doctor for at least 3 months) and non-adherence (no treatment or follow-up for at least 6 weeks) are common in the treatment of nAMD under real-life conditions in Germany. After 6 months, 79.0% of the patients did not have regular follow-ups or injections at least every 6 weeks. Only 7.5% of the patients underwent OCT examination after 3 initial dosing injections and only two OCTs had been performed in the first 12 months. The authors concluded that failings in patient management, such as restrictions in timely and adequate follow-up and retreatment, seem to be the constraining factors in Germany.

Considering a regular treatment approach, overtreatment or undertreatment of the individual patient is feared. However, the individual actual disease activity might necessitate a tailored approach with more-frequent or less-frequent IVT-AFL injections [15]. In the ALTAIR trial (NCT 02305238), patients were treated with IVT-AFL according to a TAE scheme. At the end of the first year, approximately 60% of patients had their next IVT-AFL injection scheduled at an interval of 12 weeks or more with stable VA outcomes [17, 18]. In the second year of the study, efficacy and treatment intervals were generally maintained up to and including the last assessment at week 96 [19]. Based on these results, in July 2018, the European Medicines Agency (EMA) approved the TAE scheme for IVT-AFL within the first year. These alterations were not integrated in this post hoc analysis as PERSEUS had already been closed. With this recent label extension, patients with nAMD can be treated with IVT-AFL with a proactive TAE scheme. This may offer the opportunity to treat each patient individually by identifying the particular intensity of treatment as needed, e.g., in longer than bimonthly intervals or the other way round as frequently as monthly. At best, this may lead to improved results in the stabilization of vision and simplified procedures in routine medical practice, both for patients and for doctors, optimizing patient care.

Limitations of this study include its post hoc design as well as the non-interventional structure of the PERSEUS study. As there was no predefined visit schedule, varying patient populations contributed to the observed VA data at certain time points. The strengths of this analysis are the prospective, multicenter character and large sample size of the PERSEUS study.

Viewed together, the results of this post hoc analysis underline the importance of consistent and regular treatment in order to obtain optimal outcomes and prevent recurrences, which often result in irreversible damage to retinal photoreceptors and permanent loss of VA [8, 20].

## Electronic supplementary material

ESM 1 (PDF 109 kb)
