# Supplementary material for: Importance of continuous treatment with intravitreal aflibercept injections in patients with neovascular age-related macular degeneration—12-month post hoc analysis of the PERSEUS real-world evidence study
Source: Graefes Arch Clin Exp Ophthalmol. 2020 Aug 13;259(3):601–11. doi: 10.1007/s00417-020-04803-8 (PMC7904558; doi:10.1007/s00417-020-04803-8)
Supplement: Supplementary file 1 — (PDF 109 kb) [file 417_2020_4803_MOESM1_ESM.pdf]

# Importance of continuous treatment with intravitreal aflibercept injections in patients with neovascular age-related macular degeneration – 12-month post hoc analysis of the PERSEUS real-world evidence study

Graefe's Archive for Clinical and Experimental Ophthalmology

Joachim Wachtlin, Nicole Eter, Zoran Hasanbasic, Georgios Keramas, Christine Rech, Helmut Sachs, Harald Schilling, Peter Wiedemann, Carsten Framme

Corresponding author:

Joachim Wachtlin, MD, Abteilung für Augenheilkunde, Sankt Gertrauden-Krankenhaus, Paretzer Straße 12, 10713 Berlin, Germany. Phone: +49 (0)30 8272 2425. E-mail: augenheilkunde@sankt-gertrauden.de

## Supplementary data on previously treated patients

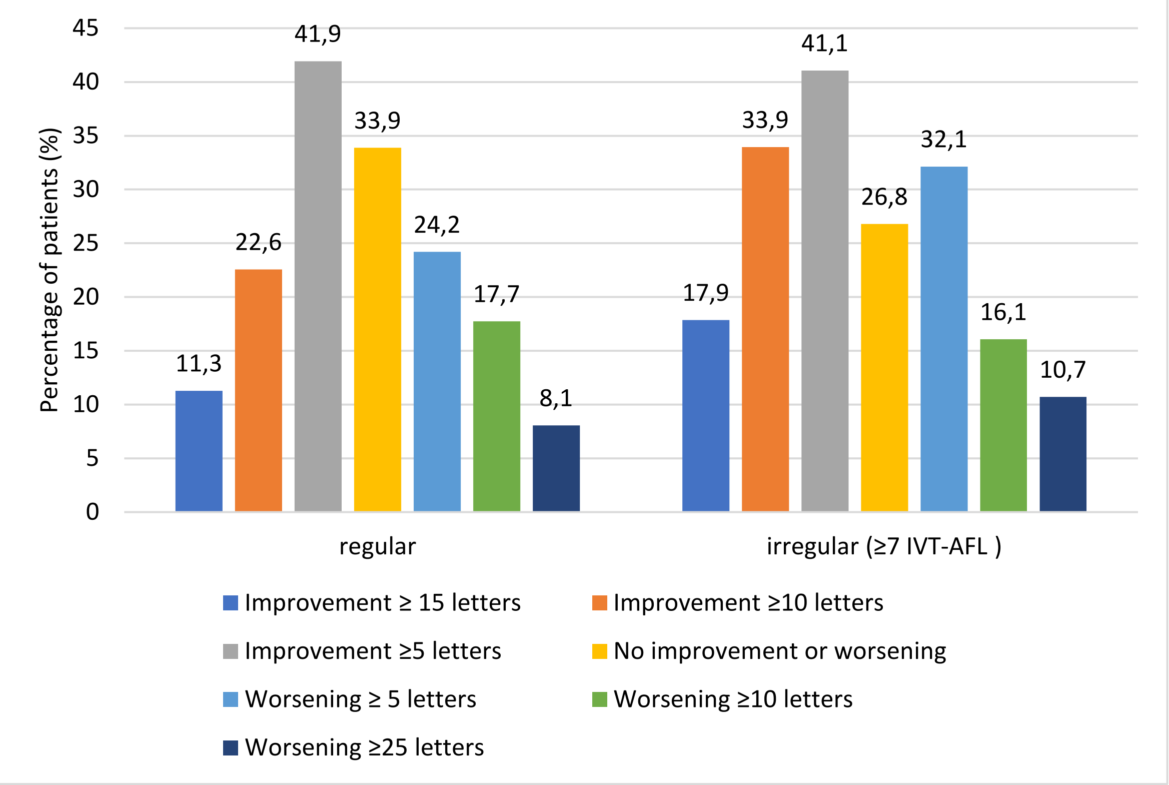


Figure 1: Proportions of patients with improvement or worsening

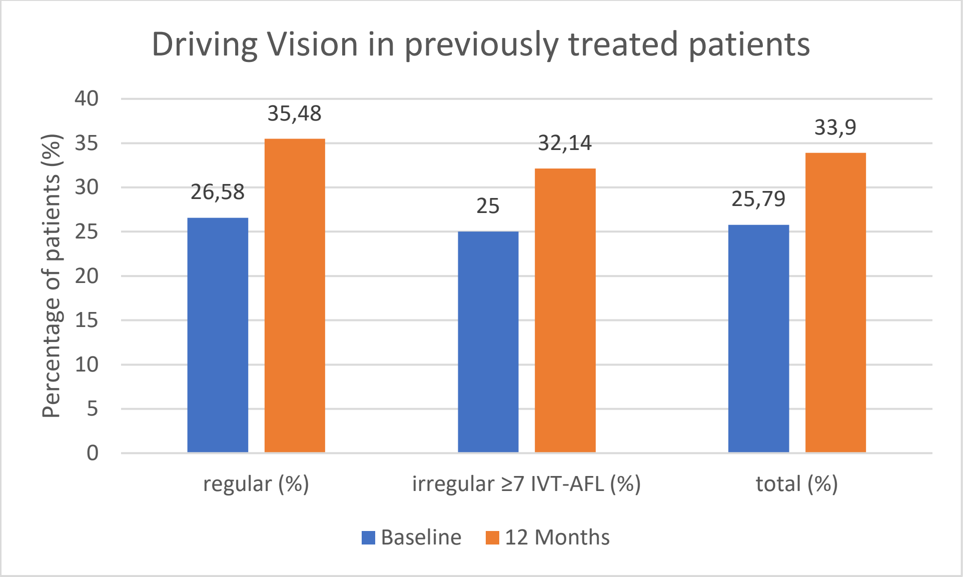


Figure 2: Proportion of patients with a VA ≥ 70 letters

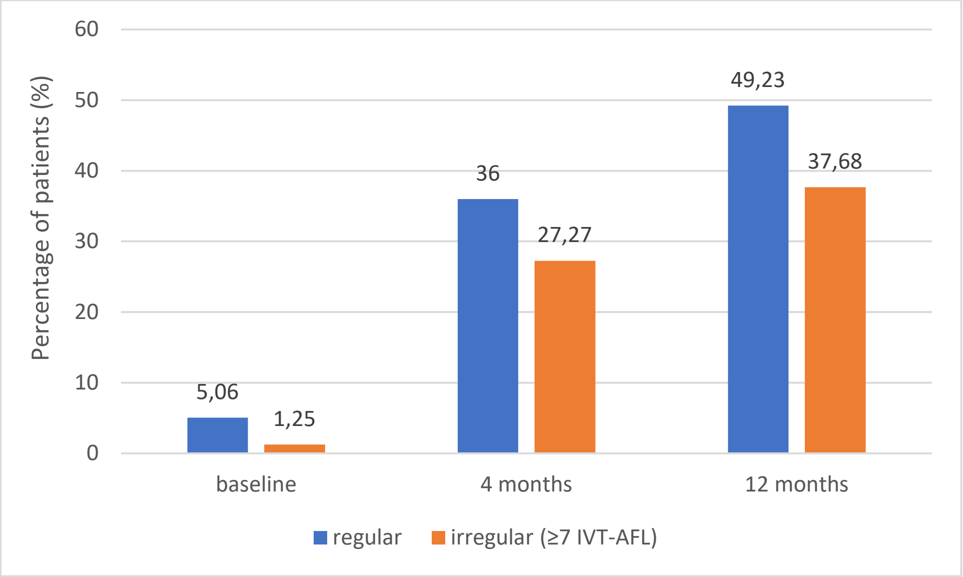


Figure 3: Proportion of patients without fluid
